# Colonic adenocarcinoma revealing Crohn's disease: a case report

**DOI:** 10.1186/1752-1947-4-167

**Published:** 2010-06-01

**Authors:** Amal Ankouz, Karim Ibn Majdoub, Abdelmalek Ousadden, Khalid Mazaz, Khalid Ait Taleb

**Affiliations:** 1 Department of General Surgery, University Hospital Hassan II of Fez, Fez, Morocco

## Abstract

**Introduction:**

There is growing evidence from epidemiological studies and clinicopathological data obtained from case reports that Crohn's disease is associated with an increased risk of carcinoma of the large bowel.

**Case presentation:**

A 70-year-old Arabic African man with undiagnosed Crohn's disease presented with acute abdominal obstruction due to an occlusive carcinoma of the sigmoid. At laparotomy, the colonic tumor was excised with continuity restored by end-to-end anastomosis.

**Conclusion:**

The risk of colonic carcinoma in Crohn's disease is increasing. Several case reports actually support the possibility that a genuine association between these two conditions exists.

## Introduction

Colorectal cancer occurring in ulcerative colitis was described in 1925 by Crohn [1] but not until 23 years later did Warren and Sommers report the first case of adenocarcinoma complicating regional enteritis [2]. For the next 30 years, an increasing frequency of reports of single [3,4] and even multiple cases [4,5] failed to dispel the scepticism surrounding this association.

## Case report

A 70-year-old Arabic African man presented to the emergency department of the University Hospital Hassan II of Fez with a five-day intestinal obstruction with associated abdominal distension and vomiting. He denied intestinal bleeding or diarrhea. He gave a history of referred intermittent episodes of constipation for a period of 6 months.

**Figure 1:**
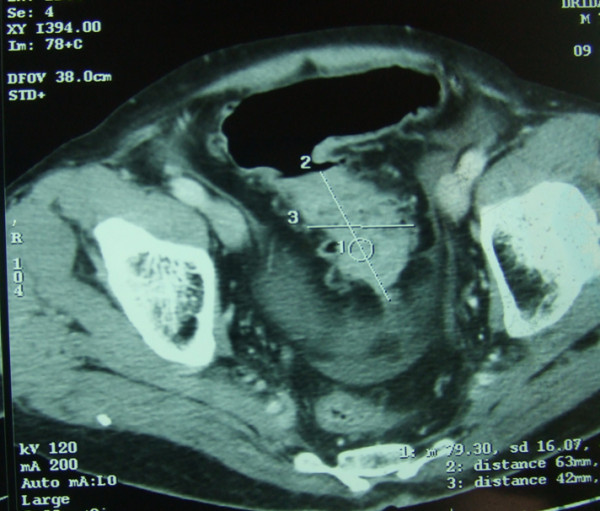
**Pre-operative abdominal scan showing the sigmoid process**.

**Figure 2:**
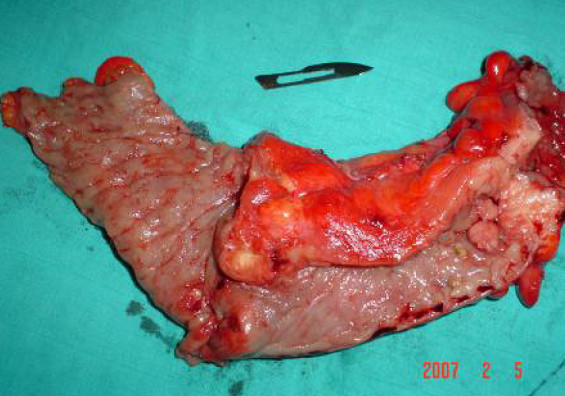
**Photograph of the surgical specimen**.

When examined he was found to have general abdominal tenderness. His white blood cell count was 8000 elt/ml, his haemoglobin was 11 gr/dl, and his platelet count was 350 k/ml. His abdominal X-rays showed air-fluid levels. Abdominal scanner examination revealed a distension of his small and large bowels upstream a sigmoid colon process (Figure 1). A sigmoidoscopy showed a stricture of his sigmoid colon. Our patient was taken immediately to laparotomy, which confirmed the presence of an occlusive sigmoid tumor. A defunctioning sigmoidostomy was later performed on our patient. A colonoscopy through the stomy revealed colitis and ileitis. A resection of his sigmoid colon was performed with continuity restored by end-to-end anastomosis (Figure 2).

**Figure 3:**
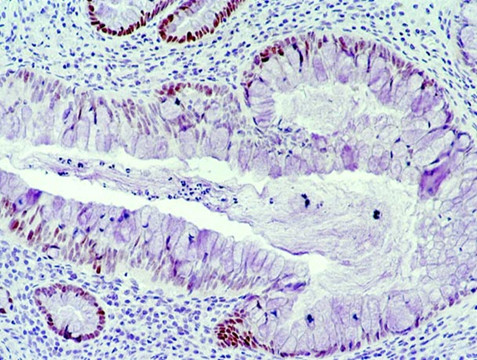
**Histological examination of the specimen revealing adenocarcinoma of the colon sigmoid**.

**Figure 4:**
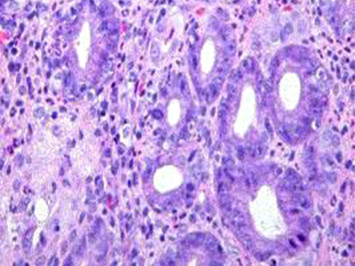
**Histology showing deep inflammation associated with pseudotuberculoid granuloma**.

Meanwhile, pathological examination of our patient showed a well-differentiated adenocarcinoma of the colon (Figure 3) arising from the areas of chronic transmural inflammation and ulcerations typical of Crohn's disease and extending through the bowel wall and invading the serosa (Figure 4). The tumor was classified T3N+M0 according to the TNM classification. Post-operative chemotherapy with 5-fluorouracil and folinic acid (5-FU+Folinic acid) was recommended but this was refused by our patient. One year later he was still well except for some episodes of diarrhea.

## Discussion

Crohn's disease is a chronic inflammatory disease whose main but non-exclusive localization is the digestive duct, more particularly the terminal ileum, colon and anus. A total of 20 cases of Crohn's disease were operated in the Digestive Surgery Department of the University Hospital of Fez from 2002 to 2007. Of the 10 patients operated in our hospital for complicated colonic Crohn's disease, we present a very unusual situation with an adenocarcinoma of the colon complicating a longstanding unknown Crohn's disease. To the best of our knowledge, there is no information on the prevalence or incidence of this association or the prevalence of Crohn's disease in Morocco. However, a north-to-south gradient could be observed.

The association between Crohn's disease and colorectal cancer has been controversial. The first reported case of colorectal carcinoma in a patient with Crohn's disease was described by Warren and Somers in 1948 [5]. Since then, an association between the two conditions has been suggested by the clinicopathological data obtained from over 150 case reports [6]. The extent of this association has been assessed by incidence studies comparing the risk of colon cancer in patients with Crohn's disease with that expected in the general population [7].

Considerable evidence supports the sequence of dysplasia and carcinoma in Crohn's disease [7-10]. Only one study has evaluated the surveillance for colorectal cancer in Crohn's disease. This study, which was undertaken in 356 patients using rectal biopsies, showed that dysplasia occurred in 5% with a predictive value of 11% for colorectal malignancy [11]. The major risk factors for the development of colorectal cancer in Crohn's disease are young age at onset, extensive disease, long disease duration, and genetic susceptibility.

Because tumor symptoms mimic those of the underlying disease, the recognition of cancer arising in an inflamed bowel becomes difficult. Thompson *et al*. [12] recognized the occult nature of intestinal cancer in Crohn's disease. They found that 59% of all cancers and 33% of colorectal carcinomas complicating Crohn's disease were discovered only at operation. Therefore, delayed diagnosis appears to be the major reason for these tumors often being diagnosed at late stages, although some reviews describe more diagnoses at early stages [13].

More colonic carcinomas in Crohn's disease are poorly differentiated and mucinous compared with sporadic colon malignancies and, overall, the prognosis is much poorer [8-10].

Surgical treatment of the disease should include a carcinological excision associated to a post-operative chemotherapy for late stages.

Evidence to date suggests that the management of colonic Crohn's disease should not be influenced by fears of malignant change. Patients with a short history should be considered as having a possible presence of both Crohn's disease and carcinoma. It should also be remembered that biopsy showing sarcoid granulomas does not exclude the presence of malignancy elsewhere.

## Conclusion

Despite the fact that the risk of colonic cancer in Crohn's disease is lower compared to that expected in the general population, vigilance is appropriate while watching over patients with inflammatory large bowel disease. The occult development of cancer in Crohn's disease makes the prognosis poorer. The advent of effective chemoprevention and the development of precocious biological markers are expected.

## Abbreviations

TNM: tumor node metastases; 5 FU: 5 fluorouracile.

## Competing interests

The authors declare that they have no competing interests.

## Authors' contributions

KM, KI and AA operated on our patient. All authors participated in the follow-up examinations of our patient and in formulating the diagnostic strategy. All authors participated in writing and revising the manuscript. All authors read and approved the final manuscript.

## Consent

Written informed consent was obtained from the patient for publication of this case report and any accompanying images. A copy of the written consent is available for review by the Editor-in-Chief of this journal.
